# Wild Mushrooms as a Source of Protein: A Case Study from Central Europe, Especially the Czech Republic

**DOI:** 10.3390/foods12050934

**Published:** 2023-02-22

**Authors:** Petr Procházka, Jana Soukupová, Kevin J. Mullen, Karel Tomšík, Inna Čábelková

**Affiliations:** 1 Department of Economics, Faculty of Economics and Management, Czech University of Life Sciences Prague, Kamýcká 129, 169 21 Prague, Czech Republic; 2 Department of Trade and Finance, Faculty of Economics and Management, Czech University of Life Sciences Prague, Kamýcká 129, 169 21 Prague, Czech Republic; 3 Department of Water Resources and Environmental Modeling, Faculty of Environmental Sciences, Czech University of Life Sciences Prague, Kamýcká 129, 169 21 Prague, Czech Republic; 4 Department of Statistics, Faculty of Economics and Management, Czech University of Life Sciences Prague, Kamýcká 129, 169 21 Prague, Czech Republic

**Keywords:** wild mushrooms, protein, consumption, cuisine, mushroom foraging

## Abstract

Wild mushroom foraging has a long tradition, especially in the region of Central Europe. Wild mushrooms are a valuable food resource, as they provide nutritional benefits to the European population. They offer a relatively high content of protein and are traditionally used in many European cuisines as a substitute for meat. This is particularly true in times of crisis, such as wars and pandemics. The study presented in this paper shows that wild mushrooms can substitute around 0.2 percent of daily protein intake and contribute around 3% to the agricultural output of the Czech economy, which was selected as a representative for Central Europe. The calculated real price of wild mushrooms indicates their increasing popularity as a source of food protein in Central Europe, while their price seems to be independent of the quantity supplied.

## 1. Introduction

Wild mushrooms, also known as mycetes, are a diverse group of fungi that grow in the wild. They can be found in a variety of habitats, including forests, grasslands, and even urban environments. These fungi play a critical role in ecosystems, serving as decomposers of organic matter and providing a source of nutrition for other organisms.

The number of species of higher fungi in Europe is around 10,000 [1,2,3,4,5], while around 700 species of edible mushrooms have been described in the literature for the region of Central Europe [2,6], and approximately 70 of them are specified as being marketable (see Appendix A). The most common types of mushrooms collected in Central Europe include boletus, chanterelles, morels, and champignons [6].

Wild mushrooms are a valuable resource for humans. They have been used for centuries as a food source and for their medicinal properties. Some species, such as the prized truffle, enjoy high prices in the market. While some wild mushrooms are safe to eat, it is important to properly identify them before their consumption [7]. In recent years, the harvesting of wild mushrooms has become a popular recreational activity. However, this can have negative impacts on ecosystems if not carried out sustainably. Over-harvesting can deplete populations of certain species, disrupting the delicate balance of the ecosystem. It is crucial to follow guidelines for sustainable harvesting, such as only taking a small portion of the population and leaving enough for the fungus to continue reproducing [8].

Wild mushrooms have long been considered a sustainable source of food due to their ability to grow in a variety of habitats and their low environmental impacts. According to [8], wild mushrooms have a low carbon footprint compared to other forms of protein. They require minimal inputs, such as water and fertilisers, and have a high yield per unit area. In addition, wild mushrooms can be harvested from forests and other natural habitats without damaging the ecosystem.

Furthermore, wild mushrooms provide important nutritional benefits. They are a good source of protein, vitamins, and minerals and have been shown to have anti-inflammatory and antioxidant properties and high water content [8].

Overall, it is important to determine the current role of wild mushrooms in protein supply in Central European countries and also look at the economic value of wild mushroom foraging. This is important because wild mushrooms can play an important role in times of crisis, provide necessary nutrition to the population, and contribute to the better health of the population. As mushrooms are becoming more popular as a marketable product within the agricultural sector, it is also necessary to look at the evolution of the prices of mushrooms. This means it is important to determine the real prices of wild mushrooms, provide predictions of wild mushroom prices for the next decade and, at the same time, calculate how much protein is supplied by wild mushrooms. This will help to describe factors that can influence wild mushroom prices over time. The uncertain political situation in the world and disease outbreak threats that may lead to decreases in protein supply and wild mushrooms may play an important role in food security.

## 2. Conceptual Framework and Overview

### 2.1. History of Wild Mushroom Harvesting and Consumption

Prehistoric humans have long considered mushrooms a common dietary enhancement. Healers, priests, and magicians probably knew the intoxicating, hallucinogenic, and sometimes even fatal or, on the contrary, healing effects of mushrooms. Extracts from poisonous mushrooms could be used in warfare or hunting. The understanding of the role of mushrooms was based on the experiences of previous generations, determined to a large extent by geographical and social conditions [9]. Clear evidence of the knowledge and use of mushrooms by Neolithic people was provided by Ötzi, a prehistoric hunter found in the ice who died about 5000 years ago. In his sachet was a slice of *fomes fomentarius* and two balls of common birch (*Piptoporus betulinus*) strung on a leather string. Perhaps they served as amulets or medicine. Wall paintings in a Chukotka cave with a completely realistic depiction of toadstools (*Amanita* sp.) are approximately the same age. They were most likely used by tribal shamans who induced ecstatic states with the help of hallucinogenic substances contained in toadstools [9]. The oldest known European depiction of edible mushrooms is on a wall fresco from Herculaneum, destroyed on August 24, 79 AD, by the eruption of Mount Vesuvius.

The culinary use of mushrooms is first mentioned in the literature of the Roman Empire. The oyster mushroom is mentioned by Marcus Apicius (first half of the 1st century AD) in the book *On the Art of Cooking* in a recipe for “mushrooms growing on trees” [9,10]. Morels also played an important role in gastronomy in Central Europe. They are among the most frequently mentioned mushrooms in Czech medieval cookbooks. Severin Mladší (1538), Bavor Rodovský from Hustiřany (1591), and others list a number of recipes for preparing morel delicacies. The recipes were purely practical: they start by washing the fruiting bodies with water to wash the sand from the mushrooms. The number of known edible mushrooms expanded over time. Julius Vincenc Krombholz mentions 33 culinarily usable species of edible mushrooms collected in nature [9]. Mushrooms were sold in markets in cities as early as the 16th century. In his herbarium, Mattioli lists 10 types of mushrooms that were consumed by people. These were mainly morels, truffles, various types of scallions, and porcini (*Boletus edulis*) mushrooms. Cookbooks from the 15th−17th centuries contain recipes for the preparation of mushrooms. In these cookbooks, mushrooms are classified as foods that replaced meat during the fasting period [10].

Today, the majority of edible mushrooms come from cultivated cultures such as oyster (*Pleurotus* sp.), various types of champignons (*Agaricus* sp.), or shiitake (*Lentinula edodes*) [11]. For more grown mushrooms, see Appendix A.

### 2.2. Protein Content and Other Nutrients

According to Kalač and Svoboda [12], the amount of protein produced in wild mushrooms in Europe varies greatly depending on the species and location. This is presented in Table 1.

Mushrooms have higher protein content than, for example, vegetables, and thus, they are considered to be an important nutrition source for many Central Europeans who prefer non-animal-based sources of protein. In particular, wild mushrooms are rich in essential amino acids and other essential nutrients, making them a valuable addition to a balanced diet [17]. Mushrooms offer important nutritional benefits and should be considered part of a healthy diet. The dry matter in mushrooms usually consists of 2–3% lipids (fats and related substances) and 8–12% minerals. The rest is made up of various carbohydrates. The low content of dry matter and lipids is the reason for the appreciated low energy value of mushrooms, usually 350 to 400 kcal per kg of fresh fruits. The abundant presence of essential amino acids in mushrooms is important for a balanced diet, with the most important one being methionine [18]. In addition to their use as a food source, wild mushrooms have also been shown to have a number of health benefits. Some species of wild mushrooms are rich in antioxidants, which can help reduce the risk of chronic diseases such as cancer [19].

### 2.3. Famines and Wild Mushrooms

Wild mushrooms have been a source of food during famines in Europe for centuries. In the Middle Ages, for example, during times of scarcity, people would venture into the forest to gather wild mushrooms for sustenance [20].

One example of this can be seen in the use of Caesar’s mushroom (*Amanita caesarea*) during times of scarcity in ancient Rome. This mushroom, which is native to the Mediterranean region, was highly prized for its delicious flavour and was often used as a substitute for meat [21]. Overall, the use of wild mushrooms as a source of food during times of famine in Europe demonstrates their importance as a valuable resource in times of need.

They are also a good source of fibre and other nutrients, making them a valuable addition to the diet during times of food scarcity [19].

One of the most famous examples of wild mushroom harvesting during a famine is the Great Famine of 1315–1317, also known as the Great European Famine. This famine was caused by a combination of factors, including a severe drought and a crop-damaging plague of insects [22]. As a result, many people were forced to rely on wild mushrooms for sustenance [20]. Between 1770 and 1772, a high consumption of mushrooms was recorded, when very rainy weather that was unfavourable for agricultural production caused the last known severe famine in Central Europe. At this time, it is recorded in the chronicles that people ate all of the crops they found in nature, including mushrooms [23]. The notes in these rare prints talk about very serious cases of famine, for example, “In Bohemia, the famine became more and more widespread and the people died constantly. Two hundred and fifty thousand people died in Bohemia during those precious years” [24].

Wild mushroom harvesting continued to be a common practice during famines in Europe throughout the centuries. In the 19th century, for example, during the Irish Potato Famine, people gathered wild mushrooms to supplement their diets [25]. In more recent times, wild mushrooms have also been used to help alleviate hunger during times of famine in Europe. For example, during World War II, the consumption of wild mushrooms was widespread in countries such as the Netherlands, where they were often the only source of food for people who were struggling to survive [26].

However, wild mushroom harvesting during famines was not without its risks. There are many poisonous species of wild mushrooms, and people who were unfamiliar with mushroom identification were at risk of consuming toxic mushrooms [22]. In recent years, wild mushroom harvesting during famines has become less common due to the availability of other food sources and improved agricultural practices for growing mushrooms artificially, such as champignons, oyster, or shiitake [20]. However, in some parts of the world, wild mushrooms continue to be an important food source during times of scarcity [25]. This is also partially true of the current armed conflict in Europe. Additionally, during the recent pandemic, wild mushrooms played an important role [27].

Overall, the history of wild mushroom harvesting during famines in Europe showcases the adaptability and resourcefulness of humans in times of need with respect to wild mushroom foraging.

### 2.4. Mushroom Foraging in Europe

Wild mushroom harvesting is a common practice in Europe, with many individuals and families engaging in this activity as a source of food and income [28].

Mushroom harvesting in Poland has been a common practice for centuries, with various species of mushrooms being collected from forests and fields throughout the country [29]. The most common species harvested in Poland include Boletus edulis, Cantharellus cibarius, and Lactarius deliciosus [5]. In recent years, there has been an increase in the demand for wild mushrooms in Poland, both domestically and internationally [30]. This has led to an increase in the number of individuals engaged in mushroom harvesting, often leading to over-harvesting and potential negative impacts on the environment [5]. To mitigate these potential impacts, the Polish government has implemented regulations on mushroom harvesting, including limits on the quantity and type of mushrooms that can be collected [31]. In addition, efforts have been made to educate the public on sustainable mushroom-harvesting practices, including leaving some mushrooms for reproduction and avoiding harvesting in protected areas [31]. Despite these efforts, there is still a need for further research and monitoring of mushroom harvesting in Poland to ensure sustainable practices are being followed and to prevent potential negative impacts on the environment [30].

Mushroom harvesting in Germany and Austria has a long history and is an important part of both countries’ economies. In Germany, the most commonly harvested mushrooms are chanterelles, boletus, and morels, while in Austria, the most commonly harvested mushrooms are boletus and chanterelles [32]. In recent years, mushroom harvesting has become more popular in both countries, with the number of mushroom pickers increasing significantly. This has been attributed to a number of factors, including a growing interest in sustainable and local food, as well as the health benefits of mushrooms, which are high in nutrients and low in calories [33].

Mushroom harvesting is a popular activity in the Czech Republic and Slovakia. These countries have a rich history of mushroom foraging, with many species of edible mushrooms growing in their forests and meadows. According to [10], the most commonly harvested mushrooms in the Czech Republic include boletus, chanterelles, and morels [10]. In Slovakia, the most popular species for harvesting are boletus, chanterelles, and porcini [34]. However, the illegal harvesting and over-collection of certain species, such as the highly prized porcini, continue to be a problem in both countries [35]. Overall, mushroom harvesting is an important cultural and economic activity in the Czech Republic and Slovakia, but it must be managed sustainably to ensure the health of forest ecosystems and the continued availability of these delicious fungi.

Mushroom harvesting in Ukraine has a long history and is an important part of the country’s agriculture and economy. In recent years, there has been an increase in the demand for wild mushrooms in Ukraine, particularly among gourmet chefs and health-conscious consumers. The country’s forests and meadows provide a diverse range of edible mushrooms, including the popular chanterelle and boletus species [36]. Wild mushrooms are now also an important substitute for meat in a time of war [10,20,26].

### 2.5. Wild Mushrooms in European Cuisine

Wild mushrooms have been a staple in European cuisine for centuries, with various species being used in dishes across the continent. Overall, wild mushrooms play a significant role in European cuisine and offer potential health benefits. Further research is needed to fully understand their potential medicinal properties.

Polish cuisine is traditional and based on national customs. Mushrooms are an inseparable part of Polish cuisine. An example is the popular bigos, which is a type of stew. There are various recipes, and typical ingredients include sauerkraut, various meats, sausage, dried mushrooms, and prunes. Mushrooms are used dried or fresh but also pickled [37].

Slovak cuisine is typically Central European, based on local ingredients. Mushrooms are mainly used in thick soups, which were sufficient as a main meal for centuries. The most famous Slovak soup is clearly “kapustnica”, which is prepared from sauerkraut, smoked meat, sausage, prunes, and mushrooms. For the case of the Czech Republic, Zibrt [38] directly recommends dishes made from wild mushrooms and notes that recipes for mushroom dishes are published in the daily press. According to Zibrt, mushrooms seem to have found greater use among the common people than among the nobility, the rich, and the gourmets. The Czech folk cuisine of the 19th century included a number of dishes made from mushrooms, which are still widely used today, such as potato, needle, or noodle mushroom soup, mushroom sauce, “kuba” made from groats with porcini mushrooms—blue mushrooms—possibly also with potatoes, mushroom porridge made from semolina with eggs, etc. [10,38].

German and Austrian cuisine is similar to Czech cuisine, where mushrooms are used as a side dish to meat and in sauces and soups. In Ukrainian cuisine, we can find pickled mushrooms as a snack, and mushroom soups and “pirizkas” are common—a traditional dish made from leavened (or other) dough filled with, e.g., meat, eggs, potatoes, cabbage, mushrooms, cottage cheese, and other fillings [37].

### 2.6. Benefits and Dangers of Wild Mushrooms

Mushrooms are an important source of protein, are a source of vitamins and minerals, and contain substances that can strengthen the immune system. Today, health-conscious consumers are increasingly looking for alternative ways to improve their diet. Mushrooms are part of a balanced diet because of their active substances, which, in the future, can prevent diseases such as diabetes, hypertension, atherosclerosis, and cancer [39].

Mushrooms have been used since ancient times to treat various diseases. Mycotherapy is based on the foundations of traditional Chinese medicine. However, only very few higher fungi have penetrated official medicine. One of them is the porcelain slug (*Oudemansiella mucida*), from which the antifungal antibiotic mucidin, later manufactured under the name Mucidermin Spofa, was isolated [40].

There are also numerous dangers associated with mushroom consumption. Seneca the Elder (55 BC–37 AD) wrote that mushrooms are “a poison that gives pleasure”. Usually, mushrooms are harder to digest, and many of them also contain substances harmful to health. For centuries, people have come to terms with this fact in a difficult and painful way. The initial knowledge of mushrooms can only be assumed to have been acquired by shamans and tribal chiefs. Unintentional poisonings certainly occurred before people learned to identify mushrooms and passed on these experiences. One of the first known mushroom poisonings is that of the family of the poet Euripides (484–406 BC). The best-known ancient story, which has been elaborated several times in the literature, is about the assassination of the emperor Tiberius Claudius Nero Germanicus (54 AD), for whom his eunuch Halótus prepared a toadstool dressing for his meal. It seems that mushrooms were also used as poison to kill unwanted persons; for example, the deaths of Pope Clement VII, King Charles VI, and Prince Beronil of Naples are attributed to mushroom poisoning [9,10,41]. Poisoning from mushrooms is also a current topic in Central Europe. For instance, Germany reports around 3000 hospitalisations and 10 deaths annually due to mushroom poisoning [41].

In addition, recently, some mushrooms were found to accumulate substances that have adverse effects on human health, especially mercury, lead, cadmium, and astatine. Therefore, it is necessary to collect edible mushrooms in non-contaminated places, outside of industrial areas, where it is certain that mushroom fruiting bodies will not contain these substances [42].

## 3. Materials and Methods

### 3.1. Questionnaire

Data regarding mushroom harvesting in the Czech Republic were taken from the publications of the Ministry of Agriculture of the Czech Republic, which are published every year under the name “Report on the State of Forests and Forestry in the Czech Republic” [43]. The reports contain summary information on the state of forests and forest management in the Czech Republic. Mushroom-harvesting data were published in the “Non-marketed Forest Production and Visits of Forest” section.

The original research used the same methodology every year. Data collection was performed within the so-called omnibus survey, a quantitative research method where the data are collected from many subjects during the same interview. The subjects were asked by trained interviewers during F2F (Face-to-Face) questioning. The process was realised through the CAPI method (Computer-Aided Personal Interview) [43].

The total number of conducted interviews was around one thousand every year. Specifically, the average number of respondents was 1019, with the lowest value being 1000 and the highest value being 1035. A representative sample was achieved using quota sampling, which examined the following factors: gender, age, education, the size of the municipality of permanent residence, and the size of the region [43]. To illustrate the optimal proportion of these factors, the following Table 2 shows the distribution of the population in the Czech Republic according to the analysed factors.

The data were collected for the years 1994–2021. There were two primary variables prepared for further analysis: (1) the annual amount of foraged wild mushrooms in the Czech Republic in mil. kg and (2) the annual collection of mushrooms in the Czech Republic expressed in current prices, in mil. CZK.

### 3.2. Prediction and Dependency

Statistical dependency was tested using regression analysis, which can be expressed using Equation (1):

(1) $${\hat{y}}_{i}=\beta_{0}+\beta_{1}x_{i}$$

where ${\hat{y}}_{i}$ is the estimated value of the dependent variable, $\beta_{0}$ is the intercept term, $\beta_{1}$ is the regression coefficient, and $x_{i}$ is the independent variable. In this study, the most common method for estimating the parameters of the regression function—Ordinary Least Squares (OLS)—was used [46].

### 3.3. Consumer Price Index

As it is important to find out the influence of inflation, the real price of wild mushrooms must be calculated. For the purpose of the real price calculation, the Consumer Price Index (CPI) was used. The Consumer Price Index measures the price of a basket containing representative goods and services in the Czech Republic. The methodology of the Czech Statistical Office uses a basket of about 450 items [47]. Based on the Consumer Price Index, it is possible to calculate the inflation rate using the following formula (Equation (2)):

(2) $$inflation\;rate=\frac{CPI_{year\;n}-CPI_{base\;year}}{CPI_{base\;year}}$$

By setting the earliest year (1994) as the base year, all values in the following years were recalculated as real prices in 1994.

### 3.4. Protein Content

Data on protein consumption were taken from the databases of ourworldindata.org [3], the World Bank Open Data [48], and the Food and Agriculture Organization of the United Nations [49]. Data sets were created that reflect protein consumption in the Czech Republic from 1994 to 2021 for annual average total protein consumption per capita, annual average plant protein consumption per capita, and annual average meat protein consumption per capita. Data on mushroom foraging were taken from the Czech Statistical Office [45].

## 4. Results

### 4.1. Protein Supply from Mushrooms

**Figure 1:**
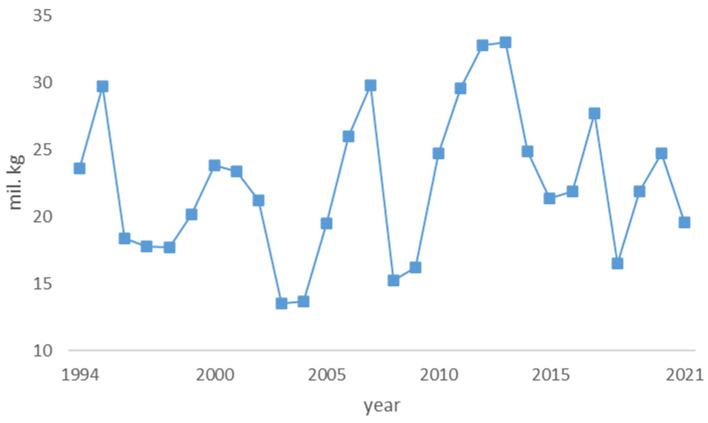
Quantity of wild mushrooms harvested in Czech forest between 1994 and 2021.

The free-of-charge collection of mushrooms is allowed on 2.5 m ha of forest area accessible to the public [45]. The quantity of mushrooms collected in the forest is depicted in Figure 1.

The figure illustrates that there are significant interannual differences in the foraging of wild mushrooms as a result of several factors of both natural and socio-economic characteristics. These differences can be also described by using descriptive statistics, which are presented in Table 3.

The data show that the median and mean are very close to each other, which implies that the data on wild mushrooms foraged are evenly distributed from the lowest to the highest values. Positive skewness indicates that there are more observations to the right of the mean. This means that there are more years with higher-than-average amounts of foraged mushrooms. The standard deviation of 5.46 indicates that the typical amount of foraged mushrooms is approximately 17 to 27 tons.

**Figure 2:**
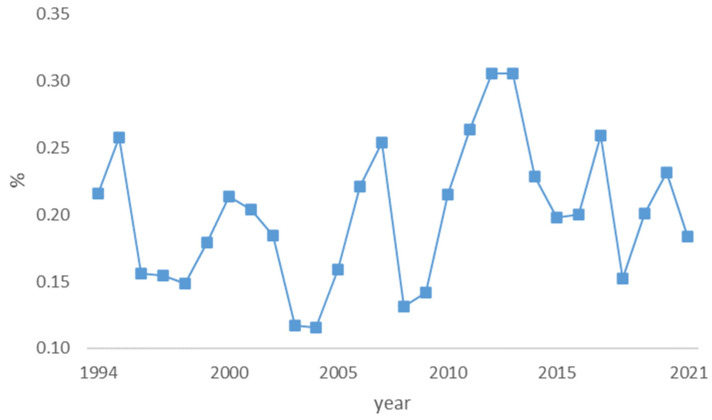
Percentage of daily per capita protein supply from wild mushrooms.

The amounts represented in Figure 1 were used as input for the calculation of the percentage of daily per capita protein supply, which is depicted in Figure 2.

It is calculated that, on average, 0.2 percent of the daily protein supply may come from wild mushrooms. Forest mushroom collection data refer to year-round collection. In the Czech Republic, mushrooms are mainly collected in the summer and autumn months, mostly from July to October. However, some types of mushrooms are also found in the winter, for example, the increasingly popular fruiting bodies of oyster mushrooms. Czechs prefer porcini mushrooms *(Boletus edulis*), but they are the most dependent on the course of the weather. They require warm temperatures and plenty of moisture in forests. While wild mushroom foraging is unpredictable in its nature, it offers an irreplaceable, sustainable, and low-cost source of protein.

### 4.2. Value of Wild Mushrooms as a Percentage of Agricultural Sector GDP

**Figure 3:**
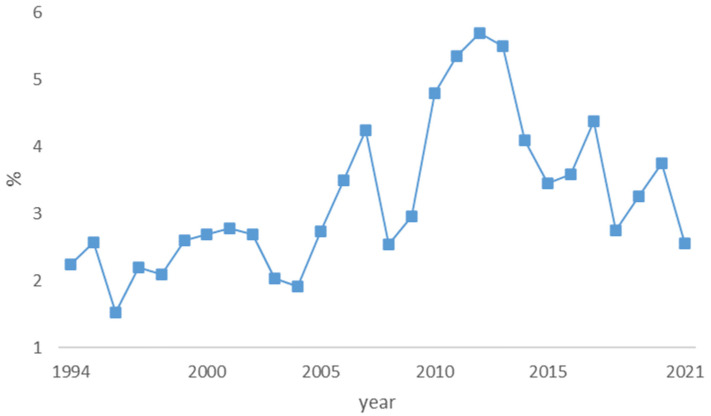
Value of wild mushrooms as a percentage of agricultural sector GDP.

As little is known about the potential value of wild mushroom foraging to the economy, the proportion of wild mushrooms foraged in the agricultural output was calculated. In Figure 3, the value of wild mushrooms as a share of the agricultural sector GDP is illustrated for the years 1994–2021. The figure shows that the wild mushroom share oscillates around 3 percent, and thus, it could significantly contribute to the agricultural gross domestic product if all mushrooms collected were sold [50,51].

### 4.3. Price of Wild Mushrooms

**Figure 4:**
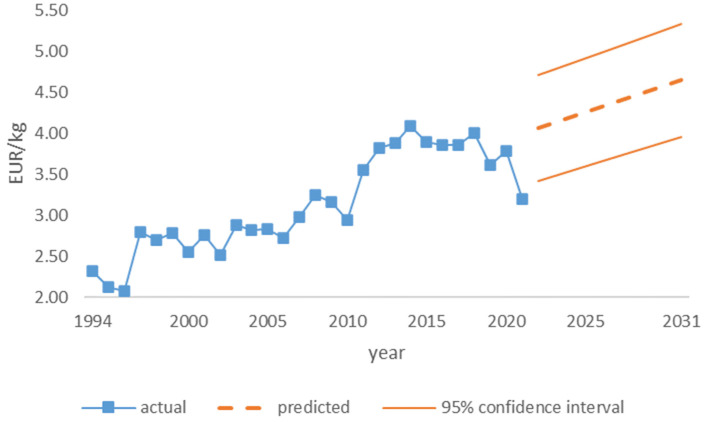
Real price of wild mushrooms (base = 1994) with prediction.

The nominal price of mushrooms was collected together with the quantity of mushrooms in the survey for every year between 1994 and 2021. The price of mushrooms was adjusted for inflation using 1994 as the base year and is represented in Figure 4, including a prediction for the next decade with a confidence interval of 95 percent [52].

The trend presented in Figure 4 is statistically significant, as presented in Table 4 below.

Therefore, it is possible to use the model for the prediction of future prices of wild mushrooms. This can be used as one of the factors that may influence the availability of protein from wild mushrooms when not foraged for free. According to consumer behaviour theory, as supply increases, the price of a good should decrease. In order to find the relationship between the quantity of foraged mushrooms and the real price, which were assumed to be stationary, regression analysis was used. The results of the regression are presented in Table 5 below.

The results indicate that wild mushrooms do not fulfil the characteristics of a commodity (like other agricultural products, such as wheat or barley), as there is no relationship between the foraged amount and the real price. A possible explanation could be that wild mushrooms are either imported or substituted in the cuisine with mushrooms that are grown and available in the Czech Republic (see Appendix A). The rising real price indicates that wild mushrooms are high in demand and will continue to play an important role as a source of nutrition, particularly protein.

## 5. Discussion

Wild mushrooms are an inseparable part of the Central European natural environment and have been collected for centuries as a unique source of food in times of both hunger and food scarcity, as well as a supplement in a diverse diet. Beranová [10] states that mushrooms were not only used in Czech cuisine in the same way as meat. They were also called the “meat of the poor” in the Czech Republic [10]. In the current difficult situation, mushrooms are a source of livelihood for some people in hard-pressed Ukraine. Many have lost their jobs and rely on mushrooms to make money and preserve food for the winter [53].

Mushrooms are an important source of protein. Chang and Buswell [54] report not only the relatively high protein content of mushrooms but also its high quality. Mushroom proteins offer all essential amino acids and are particularly rich in lysine and leucine, which are, for example, lacking in cereals [54]. The low content of fats and the high content of polyunsaturated fatty acids make mushrooms an important dietary healthy food. They are a source of vitamins, including thiamine (vitamin B1), riboflavin (B2), niacin (B3), biotin (B7), and ascorbic acid (vitamin C), as well as minerals [55]. Wild mushroom foraging therefore brings benefits in the form of healthy food to the consumer’s table. A large number of people in Central Europe are engaged in wild mushroom hunting, either for home consumption or for sale. Idnes.cz [56] states that 17.5 percent of the population of the Czech Republic clearly consider mushroom picking to be their passion, and mushroom picking is an important part of life for another 24.1 percent of people. The results of our work show that the quantity of mushrooms collected by residents does not change over time. Similarly, in other countries, the collection of mushrooms depends on their abundance in nature, which depends on natural conditions. Similar to our results, Vacik et al. [32] state that in countries where residents collect mushrooms mainly for their own consumption (Czech Republic, Germany, Austria), mushroom collection in forests is stable. In contrast, using another methodology, CSSO [57] claims that mushroom consumption per capita is increasing: in 2012, it was 2.4 kg per capita per year, while in 2021, Czechs consumed 3.3 kg per capita.

Comparing the value of mushroom foraging, the lowest quantity of wild mushrooms foraged is in Denmark, where there is also a low yield of forest mushrooms due to natural conditions. The largest amounts of foraged wild mushrooms are in Sweden (up to 38%), Poland, and the Czech Republic [58]. Research by Schulp et al. [58] further states that in 2005, 388 million kg of mushrooms was put on the market in 13 EU countries, which represents a value of EUR 169 million. However, over 90% of mushrooms are consumed for private use, so marketed mushrooms represent only a small part of the wild mushrooms foraged. This is, however, changing with the emergence of wild edible mushroom markets in some European countries, such as Finland [59]. The most popular mushrooms are chanterelles, porcini mushrooms, and morels, which are also market mushrooms [32,58].

## 6. Conclusions

The foraging of wild mushrooms has a long tradition in many parts of Europe. The popularity of wild mushroom foraging is specific to the region of Central Europe. Wild mushrooms are often used in European cuisines as a substitute for meat. Especially in times of crisis, wild mushrooms provide a nutritional source of food, especially protein. Wild mushrooms can be an important source of protein for several reasons. They are a good source of essential amino acids, which are the building blocks of protein. Additionally, wild mushrooms are low in calories and fat, making them a healthy protein option for people who are looking to maintain or lose weight. Another benefit of wild mushrooms as a protein source is that they are a sustainable and renewable resource. Unlike meat and dairy products, wild mushrooms can be harvested without harm to the environment. However, in many countries, the amounts of wild mushrooms that can be foraged are limited to mitigate environmental damage. Wild mushrooms also require less water and land to grow than many other protein sources while providing higher protein content in dry matter. The price of wild mushrooms can influence their availability. The increasing real price of mushrooms means that wild mushrooms are becoming more popular with consumers. Furthermore, wild mushrooms are often more expensive than cultivated mushrooms, which can make them less accessible to some consumers. Additionally, the harvesting of wild mushrooms can be labour-intensive, which can also drive up the cost. The conducted research shows that the real price of wild mushrooms has not been influenced by the supply of mushrooms, indicating that wild mushrooms do not possess characteristics of a commodity like other protein sources from crops such as wheat or oats. The overall contribution of wild mushrooms to the measure of agricultural output can be around three percent for the case of the Czech Republic and thus may constitute a significant source of income, particularly for rural areas.

## Figures and Tables

**Table 1:** Protein in some edible mushrooms and vegetables according to [12,13,14,15,16].

Mushrooms (Latin Name)	Crude Protein (% in Dry Matter)	Protein (g per 100 g Edible Weight)
*Boletus edulis*	26.5	5.6
*Agaricus arvensis*	56.3	2.7
*Cantharellus cibarius*	53.7	1.5
*Lactarius deliciosus*	29.8	4.4
*Morchella esculenta*	14.2	1.6
**Vegetables**	**Scientific Name**	**Protein (g)** **on Dry Weight Basis**
Cabbage	*Brassica oleracea capitata*	1.6
Cauliflower	*Brassica oleracea*	1.8
Carrot	*Daucus carota*	1.5
Lettuce	*Lactuca sativa*	1.2
Potato	*Solanum tuberosum*	1.9
Reddish	*Raphanus sativus*	1.3
Spinach	*Spinacia oleracea*	2.1
Sweet Pepper	*Capsicum annuum*	1.3
Tomato	*Lycopersicum esculentum*	0.9

**Table 2:** Representative proportions of selected factors in the Czech Republic, based on [44,45].

Factor	Category	Proportion
Gender	Female	50.7%
	Male	49.3%
Age category	18–29	14.9%
	30–39	16.5%
	40–49	20.3%
	50–59	15.9%
	60–69	15.1%
	70 and more	17.3%
Education attainment level	Primary	12.4%
	Secondary	68.2%
	Tertiary	19.4%
Size * of the municipality	Less than 200	1.6%
	200–499	6.2%
	500–999	9.2%
	1000–1999	10.3%
	2000–4999	12.7%
	5000–9999	9.2%
	10,000–19,999	9%
	20,000–49,999	12.2%
	50,000–99,999	8.5%
	More than 100,000	21%
Size * of the region	Prague (CZ010)	12.4%
	Central Bohemian Region (CZ020)	13.4%
	South Bohemian Region (CZ031)	6%
	Plzeň Region (CZ032)	5.5%
	Karlovy Vary Region (CZ041)	2.7%
	Ústí nad Labem Region (CZ042)	7.5%
	Liberec Region (CZ 051)	4.1%
	Hradec Králové Region (CZ 052)	5.1%
	Pardubice Region (CZ 053)	4.8%
	Vysočina Region (CZ 063)	4.7%
	South Moravian Region (CZ 064)	11.4%
	Olomouc Region (CZ 071)	5.9%
	Zlín Region (CZ 072)	5.4%
	Moravian-Silesian Region (CZ 080)	11%

* According to the number of inhabitants.

**Table 3:** Descriptive statistics of annual quantity of foraged mushrooms (1994–2021).

Mean	Median	Minimum	Maximum	Standard Deviation	Skewness
22.44	21.90	13.50	33.00	5.4640	0.2586

**Table 4:** Real price of wild mushrooms against time.

Variable Name	$\beta_{i}$	t-Statistic	p-Value	$r^{2}$
Constant term	2.20503	19.46	<0.0001	0.773275
Time	0.0642732	9.417	<0.0001	

Significance level α = 0.05.

**Table 5:** Real price of wild mushrooms against harvest.

Variable Name	$\beta_{i}$	t-Statistic	p-Value	$r^{2}$
Constant term	2.55703	5.283	<0.0001	0.055161
Harvest	0.0258437	1.232	0.2290	

Significance level α = 0.05.

## Data Availability

The data are available from the corresponding author.

## Appendix A. Marketable Mushrooms Species

**A. Wild Mushrooms**	**B. Artificially Grown Mushrooms**
*1. Discina perlata*	*1. Agaricus hortensis*
*2.Morchella esculenta*	*2. Agaricus brunescens*
*3. Morchella conica*	*3. Pleurotus ostreatus*
*4. Sparassis crispa*	*4. Pleurotus cornucopiae*
*5. Ramaria flava*	*5. Pleurotus pulmonarius*
*6. Hydnum repandum*	*6. Pleurotus eryngii*
*7. Cantharellus cibarius*	*7. Stropharia rugosoannulata*
*8. Cantharallus pallescens*	*8. Stropharia rugosoannulata*
*9. Craterellus cornucopioides*	*9. Flammulina velutipes*
*10. Albatrellus ovinus*	*10. Agrocybe aegerita*
*11. Albatrellus confluens*	*11. Lentinus edodes*
*12. Polyporus squamosus*	*12. Kuehneromyces mutabilis*
*13. Boletinus cavipes*	*13. Volvariella volvacea*
*14. Boletinus badius*	*14. Hirneola auricula judae*
*15. Boletus fragilipes*	*15. Pholiota nameko*
*16. Boletus luridus*	*16. Hypsizygus tessulatus*
*17. Boletus erythropus*	*17. Agaricus brasiliensis*
*18. Boletus edulis*	*18. Grifola frondosa*
*19. Boletus reticulatus*	*19. Hericium erinaceus*
*20. Boletus subtomentosus*	*20. Agaricus arvensis*
*21. Suillus variegatus*	*21.Pleurotus citrinopileatus*
*22. Suillus placidus*	*22. Pleurotus salmoneostramineus*
*23. Suillus bovinus*	
*24. Suillus luteus*	
*25. Suillus elegans*	
*26. Suillus granulatus*	
*27. Suillus aeruginascens*	
*28. Boletus scaber—Leccinum scabrum*	
*29. Boletus (Leccinum) carpini*	
*30. Boletus (Leccinum) versipelle*	
*31. Boletus aurantiacus -Leccinum aurantiacum*	
*32. Macrolepiota rhacodes*	
*33. Macrolepiota procera*	
*34. Lepista saeva*	
*35. Lepista nuda*	
*36. Tricholoma portentosum*	
*37. Calocybe gambosa*	
*38. Pleurotus ostreatus*	
*39. Pleurotus pulmonarius*	
*40. Lyophyllum decastes*	
*41. Lyophyllum fumosum*	
*42. Lactarius deliciosus (Lactarius pinicola)*	
*43. Lactarius deterrinus*	
*44. Gomphidius viscidus*	
*45. Rozites caperata*	
*46. Marasmius oreades*	
*47. Armillaria mellea*	
*48. Clitocybe nebularis*	
*49. Agaricus hortensis*	
*50. Aqaricus bitorquis*	
*51.Aqaricus campester*	
*52. Aqaricus silvaticus*	
*53. Aqaricus brunescens*	
*54. Russula heterophylla*	
*55. Russula nigricans*	
*56. Russula mustelina*	
*57. Russula vesca*	
*58. Russula cyanoxantha*	
*59. Russula viresceus*	
*60. Russula olivacea*	
*61. Russula adusta*	
*62. Russula aurata*	
